# An Exploratory Study on Resistance Spot Welding of Titanium Alloy Ti-6Al-4V

**DOI:** 10.3390/ma14092336

**Published:** 2021-04-30

**Authors:** Ichwan Fatmahardi, Mazli Mustapha, Azlan Ahmad, Mohd Nazree Derman, Turnad Lenggo Ginta, Iqbal Taufiqurrahman

**Affiliations:** 1 Department of Mechanical Engineering, Universiti Teknologi PETRONAS, Seri Iskandar 32610, Perak, Malaysia; azlan.ahmad@utp.edu.my (A.A.); iqbal_19001709@utp.edu.my (I.T.); 2 Faculty of Mechanical Engineering Technology, Universiti Malaysia Perlis, Kampus Pauh Putra, Arau 12950, Perlis, Malaysia; nazree@unimap.edu.my; 3 Orca Industri Akademi, Orca Institute for Sustainable Development, Satrio Tower, Floor 26, Unit C and D, Kuningan, Jakarta 12950, Indonesia; turnad@orca-institute.com

**Keywords:** resistance spot welding, titanium alloy, Ti-6Al-4V, welding parameter, microstructure, hardness

## Abstract

Resistance spot welding (RSW) is one of the most effective welding methods for titanium alloys, in particular Ti-6Al-4V. Ti-6Al-4V is one of the most used materials with its good ductility, high strength, weldability, corrosion resistance, and heat resistance. RSW and Ti-6Al-4V materials are often widely used in industrial manufacturing, particularly in automotive and aerospace industries. To understand the phenomenon of resistance spot weld quality, the physical and mechanical properties of Ti-6Al-4V spot weld are essential to be analyzed. In this study, an experiment was conducted using the Taguchi L9 method to find out the optimum level of the weld joint strength. The given optimum level sample was analyzed to study the most significant affecting RSW parameter, the failure mode, the weld nugget microstructure, and hardness values. The high heat input significantly affect the weld nugget temperature to reach and beyond the β-transus temperature. It led to an increase in the weld nugget diameter and the indentation depth. The expulsion appeared in the high heat input and decreased the weld nugget strength. It was caused by the molten material ejection in the fusion zone. The combination of high heat input and rapid air cooling at room temperature generated a martensite microstructure in the fusion zone. It increased the hardness, strength, and brittleness but decreased the ductility.

## 1. Introduction

Resistance spot welding (RSW) is one of the very effective welding methods used for thin or thick metal sheets. This method allows for a fast welding process and obtaining joints with good quality of strengths. Titanium alloy is one of the widely used materials in industrial needs, particularly in the automotive and aerospace industries.

Today, RSW is considered one of the most fundamental welding methods in industrial sectors and used in many production processes. The welding process is relatively simple, and the joint is generated by a heat input combined with an electrode force given to materials at a certain time. The material resistivity also plays an important role in localizing the heating area in the contact zone between the welding electrode and the material, which is then this welding method’s name taken from [1]. One of the best materials that have been used for so many years for industrial applications and welded using RSW is titanium alloy, especially Ti-6Al-4V, for example in the automotive industry. Titanium alloy grade 5 or Ti-6Al-4V has a tensile strength of 130 Ksi or 895 MPa with a yield strength of 120 Ksi or 828 MPa, making it one of the strongest alloys [2]. Titanium has low density, leading to deform easily during RSW with a high force, but in another way, it has a high melting point [3].

To define the best quality of Ti-6Al-4V weld nugget strength, it is important to investigate the weld nugget’s physical and mechanical properties. The RSW parameters such as the welding current, time, and force are proved to be the most significant affected parameters to develop the weld nugget properties [4,5,6,7,8,9,10,11,12,13,14,15,16,17,18,19,20,21,22]. Similar materials with different thicknesses were previously studied [23,24]. Some problems that occurred from the previous studies are as follows: the low maximum load in tensile-shear testing with partial interfacial failure mode was still commonly discovered, and some pores were also usually formed in the weld nugget area [23,24]. In addition, a mathematical model for an effective future maximum load and weld nugget diameter calculation has not been well studied yet. This mathematical model is very essential to increase industrial productivity and efficiency.

This study was conducted to achieve the following goals (objectives). The first objective is to determine the optimum RSW parameters to create a high maximum load in tensile-shear testing with pullout failure (PF) mode in the titanium alloy Ti-6Al-4V weld nugget. The second objective is to investigate the influence of RSW parameters into microstructure transformation. The last objective is to establish the mathematical model for the weld nugget maximum load and diameter. To achieve these objectives, several tests were conducted to do the analysis such as the tensile-shear testing, SEM, and hardness distribution testing using Vickers microhardness. Meanwhile, the mathematical model was developed using linear regression for the responses of the maximum stress and the weld nugget diameter.

## 2. Experimental Procedure

In this study, a titanium alloy (ASTM grade 5) plate with a thickness of 3 mm was used. The ASTM D1002 standard was adopted to define the sample’s geometry [25]. The AWS D8.9M:2012 standard was used to perform the welding process [26]. The experiment was taken using an AC 220V RSW machine (SL-AJ 35-600, Daiden, Osaka, Japan), which had a 14.9 kA maximum welding current and a 5.4 kN maximum welding force. The weld nugget was created using a lap joint and was welded at room temperature and common atmospheric air conditions. The welding parameters were controlled in the machine’s digital panel, and the electrode force was generated by pneumatic air pressure. The chemical composition of the titanium alloy (ASTM grade 5) is presented in Table 1.

**Figure 1:**
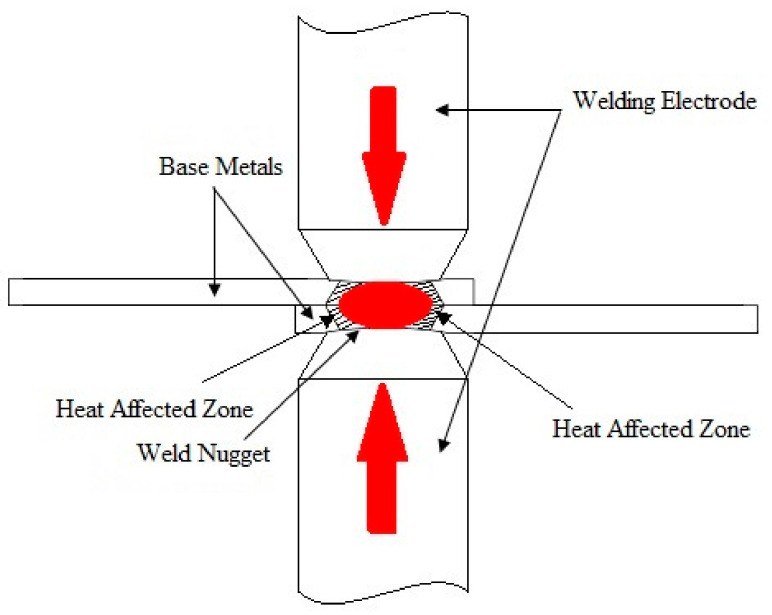
Resistance spot welding (RSW) scheme.

Figure 1 shows the RSW schematic, by using two electrodes that clamped the materials together. The heat which is created by the combination of the welding current, the welding time, and the materials resistance subsequently flows through the electrode to the materials. The weld nugget is created, when the applied heat input melts down the particular contact area between the electrode and the materials. In this study, the welding parameters were selected under the previous study consideration [23]. Due to the fact that the thickness of the titanium alloy plate (i.e., 3 mm) was three times thicker than that of the previous study material, it was considered that the welding time was increased nearly three times longer. Therefore, the welding currents were chosen to be 8, 9, and 10 kA; the welding times were chosen to be 28, 30, and 32 cycles; the forces were chosen to be 3, 4, and 5 kN. The squeeze time, the holding time, and the off-time were kept constant for 35, 15, and 0 cycles, respectively.

The Taguchi method was selected as the design of experiment (DOE) with the L9 arrangement that can be seen in Table 2. Dr. Genichi Taguchi developed the Taguchi design as a methodology set, by which at the design stage the manufacturing processes and materials’ inherent variability can be taken into account [26]. The Taguchi design is much more effective than a fractional factorial design, because the design is simply focused on orthogonal (balanced) experimental combinations and quite similar to the DOE but different from the DOE in terms of processes [4,5,6,7].

The Taguchi design proposed two distinct paths to complete the analysis. The first path was a standard approach, in which single-run results or repetitive-run averages were solved through the ANOVA (Analysis of Variance) and the main effect. The second path was multiple runs which were Taguchi prompts strongly. In this path, the analysis was carried out for similar steps but using different methods of signal-to-noise (S/N) ratios [27].

The tensile-shear test was conducted using a Zwick/Roell Universal Testing Machine (Bickenbach, Germany) with a 50 kN maximum load and a ramp speed of 20 mm/min at room temperature according to the ASTM E8/E8M-13a standard [28]. The sample’s tensile-shear testing maximum loads were analyzed using the Taguchi method in Minitab software [29]. These test results also defined the weld nugget failure mode. Microstructure observation required a cross-sectional cut of the welded joint. To obtain the cut securely the EDM, wire-cutting was conducted. The samples for microstructure analysis were etched for 10–30 s using Keller’s reagent, and this etching was based on the ASTM E407-07 standard [30]. The SEM microstructures were observed using a Zeiss scanning electron microscope according to ISO standards [31,32,33]. Hardness distribution was studied using Vickers microhardness under an indentation load of 200 gf to obtain a hardness profile according to the ASTM E92-17 standard [29,34].

## 3. Results and Discussions

### 3.1. Weld Nugget

**Figure 2:**
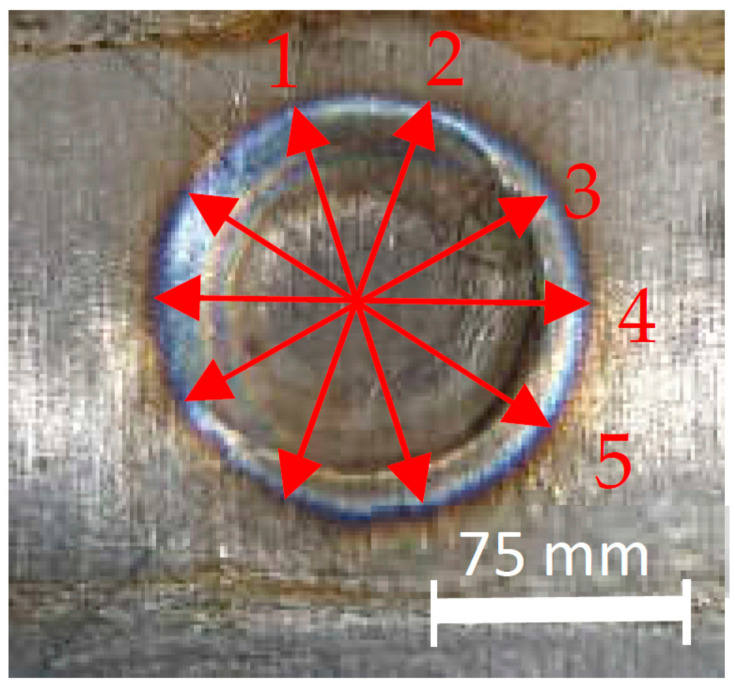
Weld nugget measurement positions.

**Figure 3:**
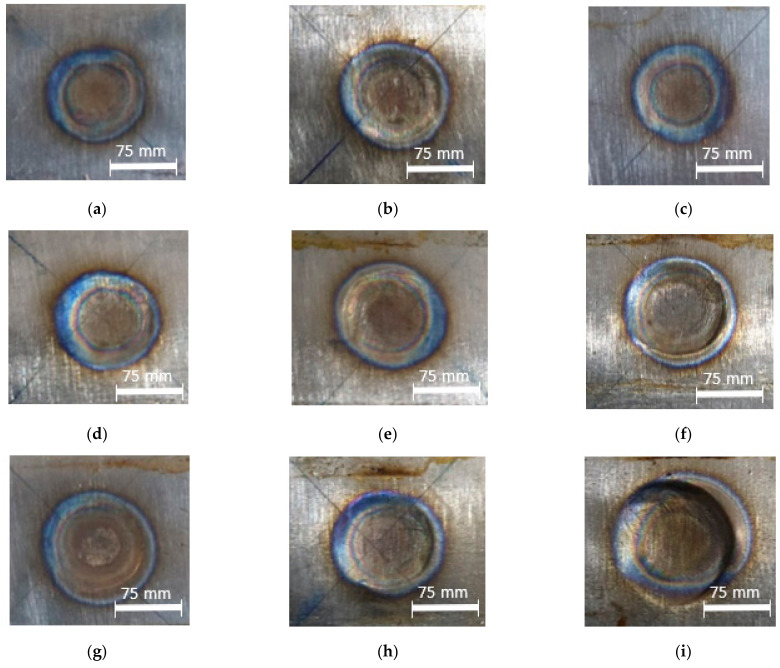
Weld nuggets at RSW parameters: (**a**) 8 kA, 28 cycles, and 3 kN; (**b**) 8 kA, 30 cycles, and 4 kN; (**c**) 8 kA, 32 cycles, and 5 kN; (**d**) 9 kA, 28 cycles, and 4 kN; (**e**) 9 kA, 30 cycles, and 5 kN; (**f**) 9 kA, 32 cycles, and 3 kN; (**g**) 10 kA, 28 cycles, and 5 kN; (**h**) 10 kA, 30 cycles, and 3 kN; (**i**) 10 kA, 32 cycles, and 4 kN.

**Figure 4:**
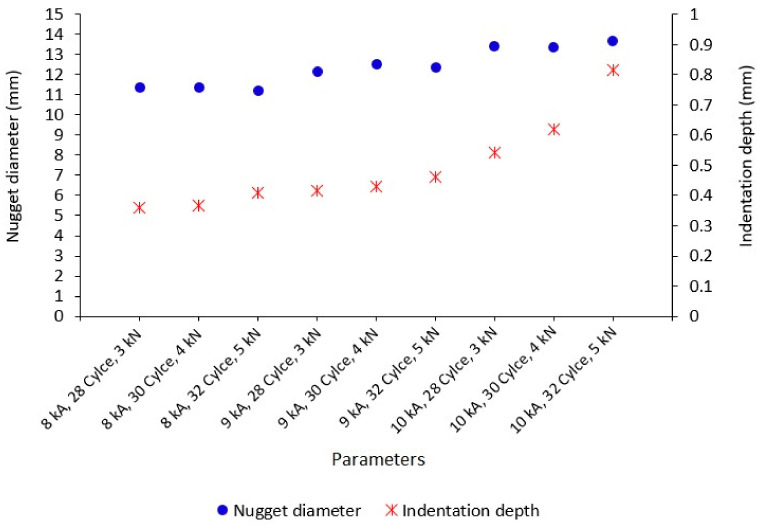
Weld nugget size.

The titanium alloy Ti-6Al-4V sheet with a thickness of 3 mm was used in this study. The materials were welded in a similar welding method. An RSW machine with an electrode diameter of 11 mm was selected and used, based on the equation described as $d>4\sqrt{t}$, where *t* is the sheet thickness [26]. To maintain the electrode’s proper condition and its resistivity after every weld, the electrode dressing was performed. This also secured the original shape and removed any scale stick upon the electrode surfaces. The measurement positions for the weld nugget diameter were taken in five positions, as shown in Figure 2. Figure 3 shows the appearance results for weld nuggets in various specimens with different RSW parameters. The weld nugget appearances for all of the specimens were completely close to the round shape, with the dark blue color on the nugget edge. It proved that the electrodes were in good condition, and the heat was generated properly. Figure 4 shows the measurements of the weld diameter and also the indentation depth from each specimen with various parameters. The indentation depths were measured using a micrometer as the difference between the base metal thickness and the fusion zone thickness divided by two.

The weld nugget growth was influenced by the combination of the heat input and the applied welding force. Initially, the clamping effect from the welding force applied from both sides of the specimens by the welding electrode kept the specimen immobile and helped localize the heat input. Then, the heat input was generated by the combination of the welding current and time, where the value was also influenced by the material and the welding electrode resistivity. It is shown in Figure 4 that the weld diameter was not significantly increased in the same welding current value, although the welding force and the welding time were increased. However, it was subsequently increased significantly, when the welding current was increased.

The heat was simply described by Joule’s law given by H = I^2^Rt [8]. From this equation, it can be drawn that weld nugget growth has a very close relationship with the heat input and its variables, particularly the welding current. The increase of the welding current will increase the heat input value subsequently [8]. At a constant specimen’s resistivity, the welding current is more influenced for the weld nugget growth, in particular the nugget diameter, than the welding time and force, because the high welding current will give a wider melting zone in the localized welding area around the weld nugget [23]. Meanwhile, the increase of the welding current and the welding time will give sufficient penetration to the weld nugget and microstructure transformation. Besides, the smaller nugget width/diameter can ensue, when a low welding force is applied, thus causing the increase of the current density and reducing the contact area in the faying layer [9]. The maximum nugget diameter will be formed, when a specific welding force, which is neither too low nor too high, is applied [9,10].

### 3.2. Taguchi Method for Tensile-Shear Testing Results and Regression Analysis

The tensile-shear test is the most common method used to evaluate mechanical properties in static conditions. Table 3 shows the results of the tensile-shear test for the whole experiment using the L9 Taguchi method. The lowest maximum load was found, when the welding parameters of an 8 kA welding current, a 28-cycle welding time, and a 3 kN welding force were found. Meanwhile, the highest stress was found, when the welding parameter of a 10 kA welding current, a 30-cycle welding time, and a 4 kN force were used. The Taguchi method analysis determined the most significant parameter and the parameter pattern which affected the maximum stress of the tensile-shear test.

**Figure 5:**
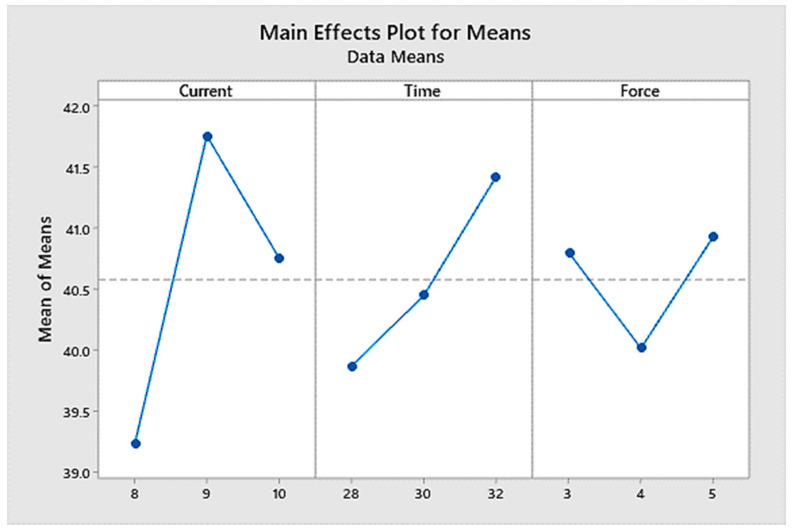
The significant effect plot of RSW parameters on the weld nugget strength.

In Table 4, it can be seen that the delta value of the welding current was the greatest of all parameters, followed by those of the welding time and the welding force. It can also be seen clearly in Figure 5 that the welding current had the most significant effect on the weld nugget strength compared to the welding time and the welding force. This is the reason that welding current ranked one as the most significant parameter which affected the maximum sample tensile load, followed by welding time ranked two and welding force ranked three, as can be seen in Table 4. A higher welding current and a higher welding time created more penetration and a denser fusion zone. The welding current of 9 kA, the welding time of 32 cycles, and the welding force of 5 kN were selected as the optimum parameters, resulting in the best maximum load, as shown in Figure 5.

**Figure 6:**
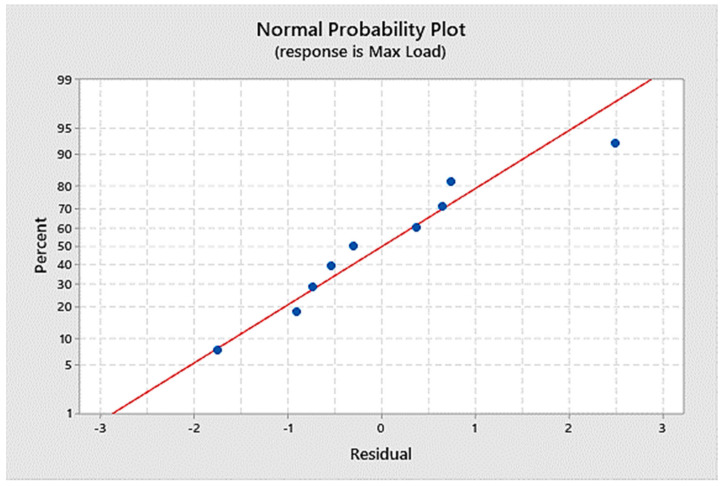
Normal probability plot as the response of the maximum Load.

**Figure 7:**
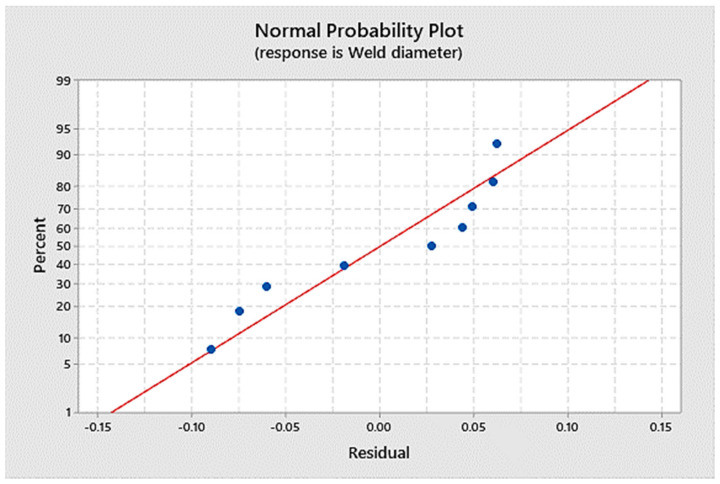
Normal probability plot as the response of the weld diameter.

Generating a mathematical model can help conduct future research. Linear regression was used to determine the mathematical model of the maximum tensile stress and weld nugget diameter as dependent variables. The welding current, the welding time, and the welding force acted as independent variables. After linear regression analysis, the ANOVA with an adjusted R-square of 94.68% is given in Table 5. Figure 6 shows the normal probability plot for the maximum stress, and Figure 7 shows weld diameters as the responses. Thus, the regression equation for mathematical models were given as:

Max Load = 21.8 + 0.763 Current + 0.389 Time + 0.064 Force(1)

Weld diameter = 0.545 + 1.0869 Current + 0.0665 Time + 0.0114 Force(2)

**Figure 8:**
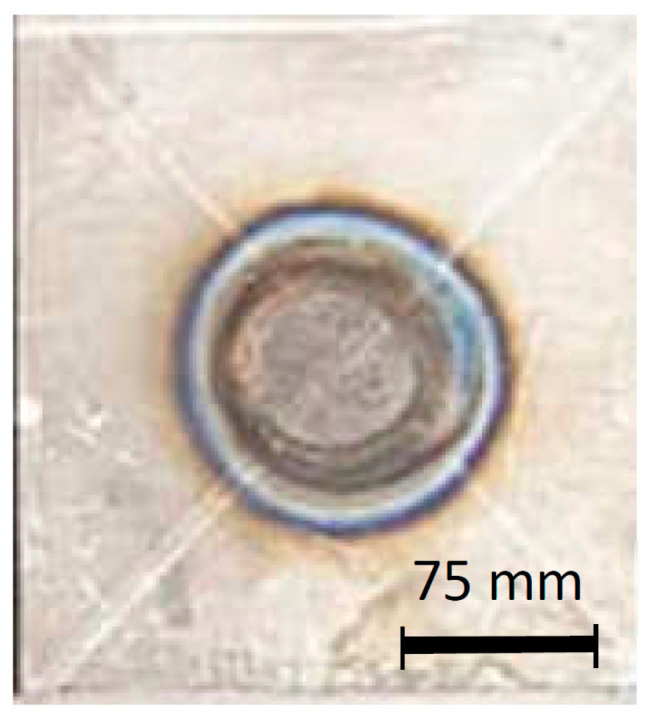
The sample weld nugget appearance by the confirmation test.

**Figure 9:**
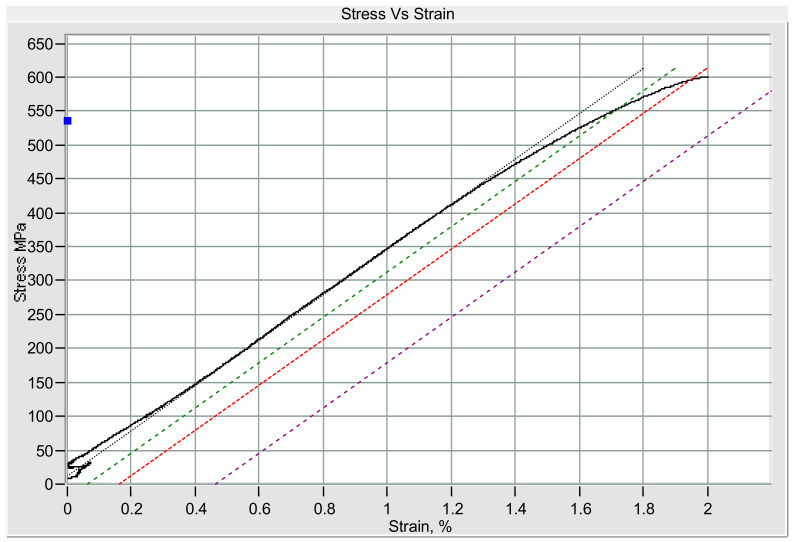
Stress and strain relationships of the samples obtained by the confirmation test.

A confirmation test was taken from the selected optimum parameters result, which was given by the ANOVA. From Figure 8, it is known that the weld nugget shape was round. The average diameter measurement, which was taken from six circular positions, was 12.477 mm. The measured indentation depth was 0.45 mm with no expulsion appearing. The values of the tensile-shear result are displayed in Table 6, showing that the maximum stress of 599.724 MPa and the maximum load of 44.979 kN, which is the highest value among all of the results given in the Taguchi Table 3. As shown in Table 6 and Figure 9, the yield strain was 1.947%, which was high enough to withstand high stress.

### 3.3. Failure Mode

In the weld nugget physical properties analysis, the failure mode and the fracture mechanism are also important components. PF, interfacial failure (IF) partial interfacial mode (PIF), and partial thickness-partial pullout (PT-PP) mode are the most common failure modes in tensile-shear testing results [14,15,16,17,18,19,20,21]. The weld nugget failure mode is influenced by the fusion zone dimension, the sheet thickness, and the weld nugget hardness-to-failure location hardness ratio during tensile-shear testing [20].

**Figure 10:**
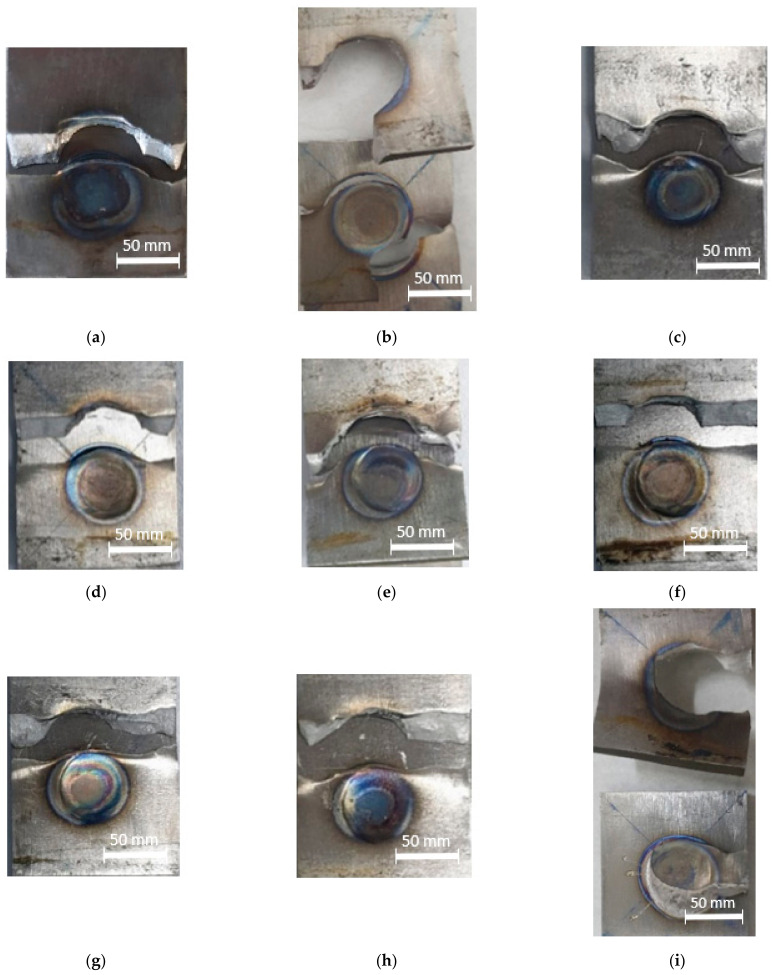
Weld nuggets failure modes at RSW parameters: (**a**) 8 kA, 28 cycles, and 3 kN; (**b**) 8 kA, 30 cycles, and 4 kN; (**c**) 8 kA, 32 cycles, and 5 kN; (**d**) 9 kA, 28 cycles, and 4 kN; (**e**) 9 kA, 30 cycles, and 5 kN; (**f**) 9 kA, 32 cycles, and 3 kN; (**g**) 10 kA, 28 cycles, and 5 kN; (**h**) 10 kA, 30 cycles, and 3 kN; (**i**) 10 kA, 32 cycles, and 4 kN.

As can be seen in Figure 10a, it is shown that the failure mode was PIF. The low welding current contributed to low heat input and the generation of a fusion zone with a low strength, thus resulting in IF mode in the fusion zone [22], because the localized heating only occurred in the small area covered by the welding electrode and resulted in a low heating temperature. It is also related to the generation of a smaller weld nugget diameter and a shallow indentation depth, and the transformation of the microstructure. This created a brittle fracture in the fusion zone after the tensile testing, indicated by the lowest maximum load in Table 3. Figure 10b shows that the failure mode was still the PIF but the fracture started to move away from the fusion zone and hit the heat-affected zone (HAZ).

A higher welding current helped increase the weld nugget size and the indentation depth, and created a different microstructure, then causing the maximum load to increase. In Figure 10c–f, the failure modes were changed into PF. The HAZ was seemingly more favorably affected than the fusion zone, except for that shown in Figure 10f. The fracture location in Figure 10f was observed to occur in the base metal, leaving the fusion zone and the HAZ undamaged. In Figure 10g,h, the failure modes were still PF, but the maximum loads were decreased. Particularly in Figure 10i, the failure mode changed into a PI-PP with expulsion appearing. The expulsion caused material degradation, so that the fusion zone thickness became thinner. This event lowered the weld nugget strength indicated by the decrease of the maximum load during the tensile-shear testing.

**Figure 11:**
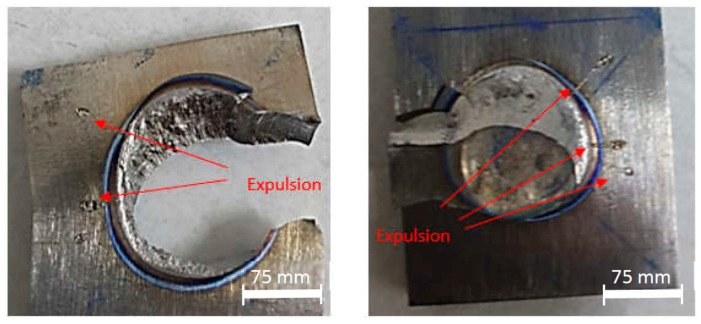
Expulsion under the conditions of a welding current of 10 kA, a welding time of 32 cycles, and a welding force of 4 kN.

**Figure 12:**
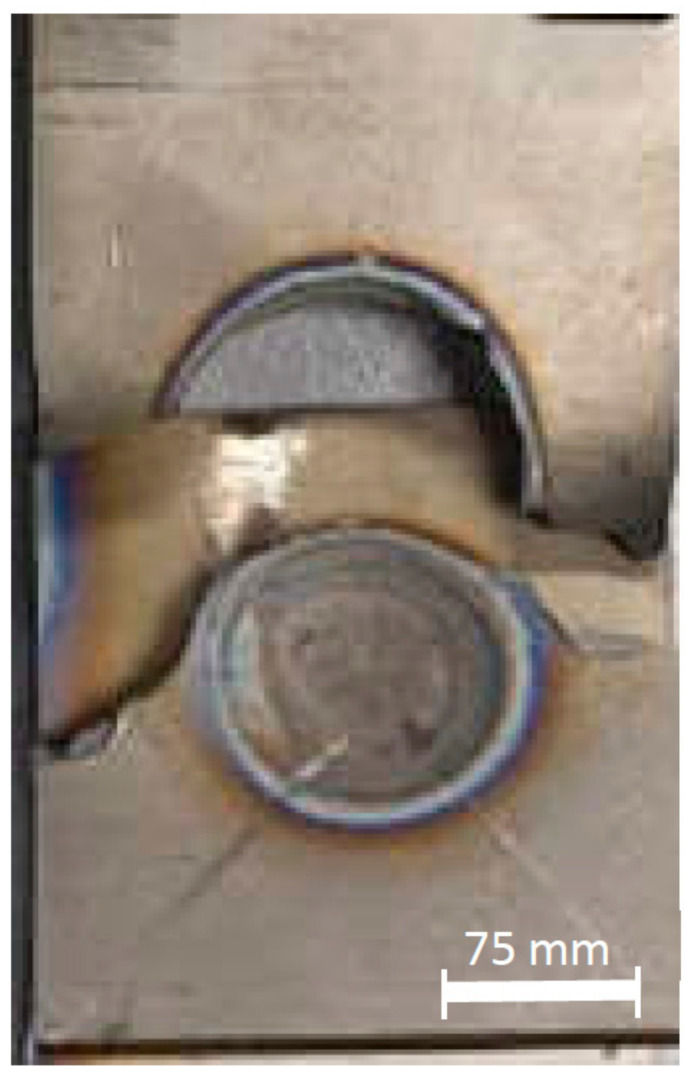
The sample tensile-shear testing failure mode observed by the confirmation test.

An expulsion can be explained as following: when a higher welding current was given which developed a higher heat input in a short welding time, an expulsion was generated. This expulsion was caused by overheating and degrading the material deposit to the air suddenly [35,36]. The expulsion created a higher indentation depth in the area which included the fusion zone and the HAZ as well. The significant impact of the expulsion event affected the weld nugget final yield strength. Therefore, the expulsion event is one of the most essential factors to detect and characterize the weld nugget quality assurance [19]. The possible outcome of the expulsion formation can also be generated, when the electrode displacement exceeds the limit of the maximum expansion, leading to an explicit spatter formation [11]. Consideration can be taken, as the expulsion tends to increase when a too low or too high welding force is applied [10]. As can be seen in Figure 11, there was a slight expulsion on the side of the weld nugget, and therefore, the indentation depth increased significantly, as shown in Figure 4. A massive expulsion created a bigger nugget diameter but with a higher indentation depth. From Figure 12, the appearance of weld nugget failure after tensile-shear testing can be seen. It is shown that the failure mode was still the PF mode mostly in the HAZ and the base metal with no expulsion appearing both in the outside and the inside of the weld nugget.

### 3.4. Microstructure

**Figure 13:**
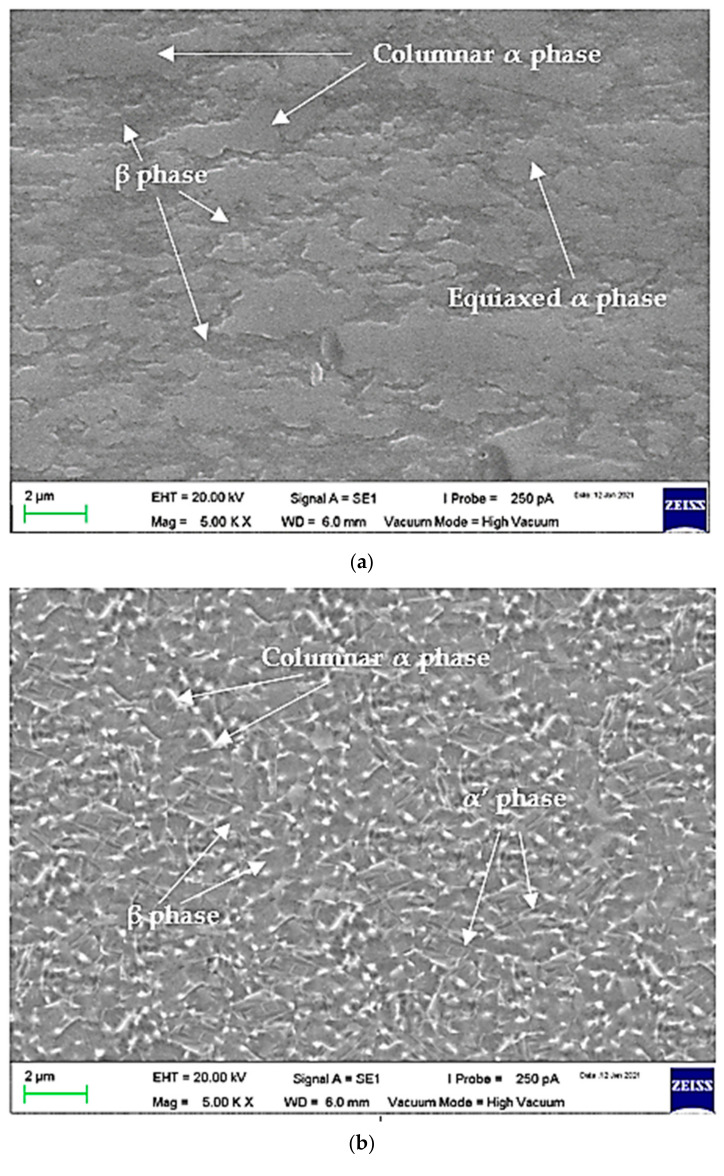

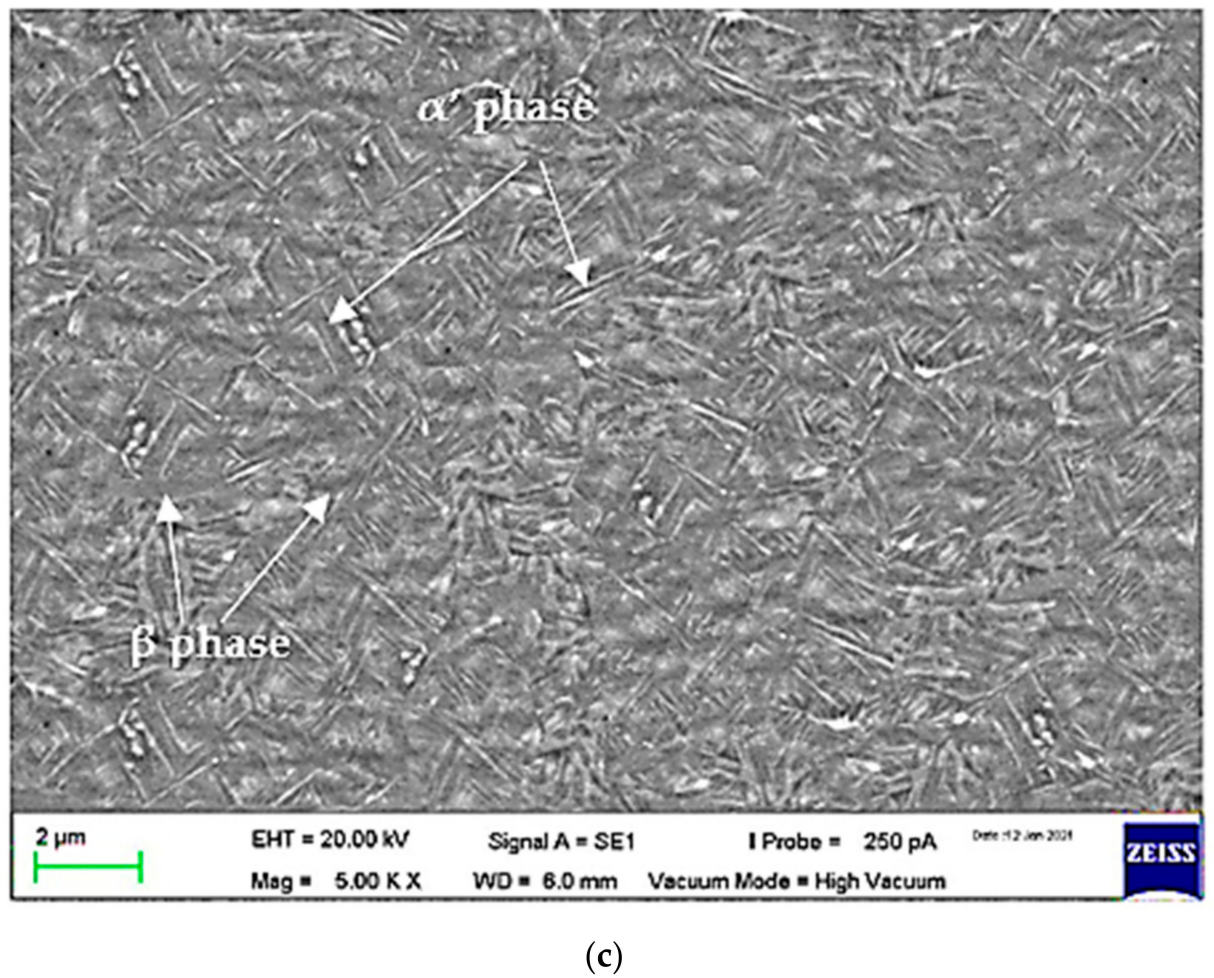
The sample SEM images at different positions by the confirmation test: (**a**) base metal; (**b**) heat-affected zone; (**c**) fusion zone. Magnification: 5000×.

The microstructure of Ti-6Al-4V consists of the α, β, or α + β phase. Ti-6Al-4V with the α phase has good strength, toughness, and weldability but has poor forgeability. Meanwhile, Ti-6Al-4V with the β phase is an alloy composed of vanadium, niobium, and molybdenum, which has good forgeability over a wide temperature range [3]. The presence of this alloy is also useful to reduce the temperature transition of the α phase to the β phase, thereby contributing to the formation of a β-phase body-centered cubic (BCC) structure. Figure 13 shows the SEM image of the BCC structure with a 5000× magnification at three positions, namely base metal, HAZ, and fusion zone. Figure 13a shows the base metal microstructure was duplex which consisted of the α phase (light color) and β phase (dark color) and mostly consisted of columnar α and equiaxed α. Figure 13b shows that the β-phase stabilizer which had a BCC structure transformed into the α’ phase which had a hexagonal close-packed (HCP) structure at the β transus temperature around 980–1000 °C, spread over the HAZ and began to multiply in the fusion zone, as shown in Figure 13c [4].

The α’ phase is known as the supersaturated solid solution of elements in the Tiα phase, which is transformed in a β phase stability temperature range at a rapid cooling rate [37]. At this temperature, a lot of β phases transform into α’ phrases. Both α and α’ phases have HCP structures [38]. The high heating temperature in the fusion zone and the rapid cooling rate (air cooling) that is evenly distributed in the fusion zone compared to in HAZ greatly contributed to many transformations of the β phase into the α’ phase. The high temperature spread from the contact center of the electrode with the material radially towards the base metal, so that even in a thin HAZ, the α’ phase was observed. This would rather coarsen the grain in the fusion zone than in the base metal, which had a lower temperature.

**Figure 14:**
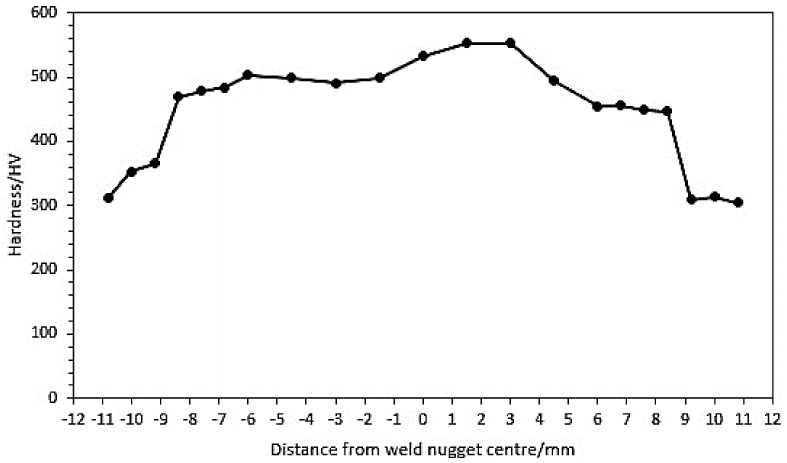
Vickers microhardness test results with the optimum parameters.

The Vickers microhardness test was performed on a cross-sectional area of the weld nugget at 25 points starting from the base metal towards the fusion zone, according to Figure 14. The results of the Vickers microhardness test are shown in Figure 14. It can be seen that the base metal with a duplex microstructure provided the lowest hardness value of the overall test results. With the testing getting closer to the fusion zone, the observed hardness values were increased, starting in the HAZ with the acicular α’ martensitic population which began to form a lot. The martensitic acicular α’ phase can cause an increase in the hardness value. The attainment of β-transus temperature by rapid cooling (air cooling) with a long welding time caused changes to occur. This microstructure transformed the structure from the BCC structure to become the HCP structure, which had a vast increase in volume, generating a highly stressed structure. That is the reason why the martensite microstructures had higher hardness and higher strengths in the fusion zone and the HAZ, which than in the base metal but less ductile and too brittle.

## 4. Conclusions

A comprehensive investigation of Ti-6Al-4V weld nugget physical and mechanical properties was conducted, and the conclusions are as following:

1. The higher welding current increased the heat input significantly, which caused the wider melting zone in the faying layer of the contact area between the electrode and the sample. The weld nugget diameter and indentation depth tended to grow higher subsequently.
2. The tensile testing maximum load was increased following the increase of heat input, but an expulsion was generated when the heat input highest level was achieved and the melted zone was overheated at a short welding time and ejected some material deposits instantly in the air.
3. The heat input generation was affected significantly by the welding current followed by the welding time and the welding force. In the regression analysis, the response table for means shows the highest delta value with the medium level of welding current of 9 kA as the selected optimum level. This was supported by the highest stage with a welding time of 32 cycles and a welding force of 5 kN, which also created a dense fusion zone. Meanwhile, the lowest welding current created a colder weld. Otherwise, the highest welding current generated expulsion, although at this level a deeper penetration was achieved.
4. The weld nugget strength reduction occurred in the highest stage and the lowest stage of heat input. These were indicated by the low maximum load results. In these levels, a brittle fracture also attacked the HAZ and the fusion zone. The selected optimum RSW parameters gave the highest tensile-shear testing result with no expulsion appearing in the weld nugget.
5. When a higher heat input was applied, a larger martensitic microstructure with the α’-phase presence as the transformation from the β phase in β-transus temperature was formed with no pores appearing at all. This structure as a highly stressed structure was HCP, causing the fusion zone to have the highest hardness value among all areas.

## Figures and Tables

**Table 1:** Chemical compositions of the titanium alloy ASTM grade 5 [2].

Element	Composition (%)
Nitrogen (max)	0.05
Carbon (max)	0.08
Hydrogen (max)	0.015
Iron (max)	0.40
Oxygen (max)	0.20
Aluminum	5.5–6.75
Vanadium	3.5–4.5
Titanium	Balance

**Table 2:** Experimental design using the Taguchi method.

Experiment	Welding Current (kA)	Welding Time (Cycles)	Welding Force (kN)
1	8	28	3
2	8	30	4
3	8	32	5
4	9	28	4
5	9	30	5
6	9	32	3
7	10	28	5
8	10	30	3
9	10	32	4

**Table 3:** Maximum load results of the tensile-shear testing.

Experiment	Welding Current (kA)	Welding Time (Cycles)	Welding Force (kN)	Max Load (kN)
1	8	28	3	38.061
2	8	30	4	39.51
3	8	32	5	40.111
4	9	28	4	40.167
5	9	30	5	41.295
6	9	32	3	43.795
7	10	28	5	41.366
8	10	30	3	40.532
9	10	32	4	40.361

**Table 4:** Response for means.

Experiment	Welding Current (kA)	Welding Time (Cycles)	Welding Force (kN)
1	39.23	39.86	40.80
2	41.75	40.45	40.01
3	40.75	41.42	40.92
Delta	2.53	1.56	0.91
Rank	1	2	3

**Table 5:** ANOVA.

Source	DF	Adjusted SS	Adjusted MS	F-Value	*p*-Value
Regression	3	7.156	2.385	0.97	0.474
Current	1	3.491	3.491	1.43	0.286
Time	1	3.639	3.639	1.49	0.277
Force	1	0.025	0.025	0.01	0.924
Error	5	12.240	2.448	-	-
Total	8	19.396	-	-	-

**Table 6:** Confirmation test results of the sample tensile-shear testing results.

Peak Stress (MPa)	Peak Load (kN)	0.2% Offset Yield Stress (MPa)	Yield Strain (%)	Yield Load (kN)	0.02% Offset Yield Stress (MPa)	Modulus (GPa)	0.1% Offset Yield Stress (MPa)
599.724	44.979	595.68	1.947	44.676	461.483	33.409	553.845

## Data Availability

The data presented in this study are available on request from the corresponding author.
